# Nutritional, functional, and bioactive properties of african underutilized legumes

**DOI:** 10.3389/fpls.2023.1105364

**Published:** 2023-04-14

**Authors:** Jacob Olagbenro Popoola, Omena B. Ojuederie, Oluwadurotimi Samuel Aworunse, Aminat Adelekan, Abiodun S. Oyelakin, Olusola Luke Oyesola, Paul A. Akinduti, Samuel Olatunde Dahunsi, Taofeek T. Adegboyega, Solomon U. Oranusi, Modupe S. Ayilara, Conrad A. Omonhinmin

**Affiliations:** ^1^ Pure and Applied Biology Programme, College of Agriculture, Engineering and Science, Bowen University, Iwo, Osun, Nigeria; ^2^ Department of Biological Sciences/Biotechnology Cluster, Covenant University, Ota, Ogun, Nigeria; ^3^ Department of Biological Sciences, Kings University, Ode-Omu, Osun, Nigeria; ^4^ Food Security and Safety Focus, Faculty of Natural and Agricultural Sciences, North-West University, Mmabatho, South Africa; ^5^ Department of Chemical and Food Sciences, College of Natural and Applied Sciences, Bells University of Technology, Ota, Ogun, Nigeria; ^6^ Department of Pure and Applied Botany, College of Biosciences, Federal University of Agriculture, Abeokuta, Nigeria; ^7^ Microbiology Programme, College of Agriculture, Engineering and Science, Bowen University, Iwo, Osun, Nigeria; ^8^ The Radcliffe Institute for Advanced Study, Harvard University, Cambridge, MA, United States; ^9^ Biology Unit, Faculty of Science, Air Force Institute of Technology, Kaduna, Nigeria

**Keywords:** antioxidants, bioactive compounds, functional food products, under-exploited legumes, sustainable development

## Abstract

Globally, legumes are vital constituents of diet and perform critical roles in maintaining well-being owing to the dense nutritional contents and functional properties of their seeds. While much emphasis has been placed on the major grain legumes over the years, the neglected and underutilized legumes (NULs) are gaining significant recognition as probable crops to alleviate malnutrition and give a boost to food security in Africa. Consumption of these underutilized legumes has been associated with several health-promoting benefits and can be utilized as functional foods due to their rich dietary fibers, vitamins, polyunsaturated fatty acids (PUFAs), proteins/essential amino acids, micro-nutrients, and bioactive compounds. Despite the plethora of nutritional benefits, the underutilized legumes have not received much research attention compared to common mainstream grain legumes, thus hindering their adoption and utilization. Consequently, research efforts geared toward improvement, utilization, and incorporation into mainstream agriculture in Africa are more convincing than ever. This work reviews some selected NULs of Africa (Adzuki beans (*Vigna angularis*), African yam bean (*Sphenostylis stenocarpa*), Bambara groundnut (*Vigna subterranea*), Jack bean (*Canavalia ensiformis*), Kidney bean (*Phaseolus vulgaris*), Lima bean (*Phaseolus lunatus*), Marama bean (*Tylosema esculentum*), Mung bean, (*Vigna radiata*), Rice bean (*Vigna Umbellata*), and Winged bean (*Psophocarpus tetragonolobus*)), and their nutritional, and functional properties. Furthermore, we highlight the prospects and current challenges associated with the utilization of the NULs and discusses the strategies to facilitate their exploitation as not only sources of vital nutrients, but also their integration for the development of cheap and accessible functional foods.

## 1 Introduction

Legumes are a group of flowering plants and are classified under the Fabaceae family. This family is the third-largest in terms of angiosperm groups, consisting of over 800 different types and around 20,000 species. Within the Fabaceae family, there are three subfamilies known as Papilionoideae, Caesalpinioideae, and Mimosoideae. Of these, the edible legumes are grouped in the sub-family Papilionoideae. Globally, legumes are regarded as a valuable and inexpensive alternative protein sources and rank second after cereals as the most important food crop (Maphosa and Jideani, 2017). Apart from the rich protein and amino acid content, legume seeds provide a substantial amount of carbohydrates, minerals, and vitamins (Vadivel et al., 2012; Barman et al., 2018). In addition to having no cholesterol and gluten, legumes possess low fat and glycemic index and are rich in dietary fiber and antioxidants. These legumes possess bioactive compounds which possess antidiabetic, antimicrobial, anti-atherogenic, anti-thrombogenic, anti-hypertensive, and anticancer properties amongst others. Legumes also serve as fodder for livestock and fix atmospheric nitrogen in soils, thereby enhancing soil fertility and invariably promoting agricultural sustainability. They are also adapted to diverse agro-ecological zones and unfavorable environmental conditions, possessing structures for augmenting the sustainability of dry subtropical and tropical agricultural systems (Khoury, 2015).

**Figure 1:**
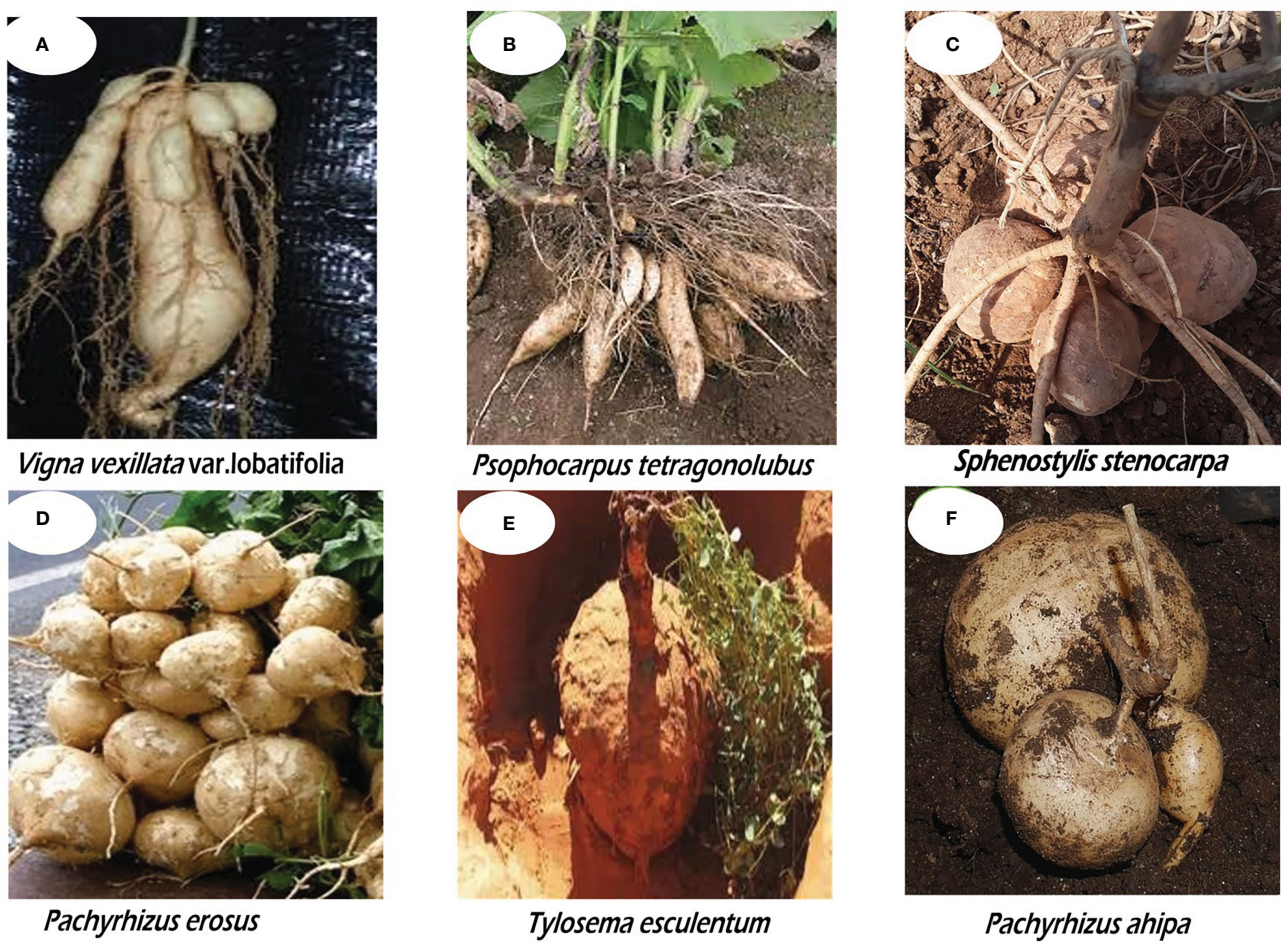
Different tuber shapes and sizes of some underutilized legumes: **(A)** Zombi pea (*Vigna vexillata*), **(B)** Winged bean (*Psophocarpus tetragonolobus*), **(C)** African yam bean (*Sphenostylis stenocarpa*), **(D)** Mexican yam bean (*Pachyrhizus erosus*), **(E)** Marama bean (*Tylosema esculentum*), **(F)** Ahipa (*Pachyrhizus aphipa*).

It is interesting to note that some legumes also produce underground tubers in addition to edible seeds. However, only a few of these legumes are incorporated into the human diet. Such dual food legumes fall into the category of neglected and underutilized legumes (NULs) simply because they have not received much research focus and are still cultivated at the subsistence level by resource-poor farmers who hold the genetic resources of these plants. Tuberous underutilized legumes are gradually gaining recognition. These include the African yam bean (AYB) (*Sphenostylis stenocarpa*) cultivated in West Africa for the seeds and in East and Central Africa for the tubers (Adewale and Nnamani, 2022); winged bean (*Psophocarpus tetragonolobus*), grown and cultivated in Papua New Guinea Highland, northern Ghana, and Burma; the Marama bean (*Tylosema esculentum*) cultivated in the Southern Africa regions of Botswana, Namibia, Mozambique, Zambia, and in northern South Africa (Abberton et al., 2020a; Abberton et al., 2020b; Ojuederie et al., 2021; Sriwichai et al., 2021); Mexican yam bean (*Pachyrhizus erosus*); Zombi pea (*Vigna vexillata*) an underutilized legume with a pantropical distribution; hyacinth bean (*Lablab purpureus*) grown in North Africa; as well as Tala (*Neoapaloxylon tuberosum*) cultivated in Madagascar (Von Wettberg et al., 2021). Different tuber shapes and sizes of some tuberous underutilized legumes are presented in **Figure 1**.

The Bambara groundnut (*Vigna subterranea*) is a crop that is extensively grown for its seeds in certain regions of West and Southern Africa. Nigeria has been reported to be the largest producer of this crop (Ojuederie et al., 2021; Popoola et al., 2022b; Arise et al., 2022). Tubers of Zombi peas are crispy, rich in protein (15%) and can be consumed raw (Tripathi et al., 2020; Von Wettberg et al., 2021). The seeds and tubers of many of the NULs are also rich in protein. For instance, AYB seeds contain 19.5% protein, while the tubers hold about 15.5% protein (Ojuederie and Balogun, 2017; Ojuederie and Balogun, 2019; Abberton et al., 2020a). In winged bean, the protein content of the seeds and tubers are 29.8% to 42.5% and 20% respectively (Abberton et al., 2020b). Negi and Gaur (1994) stated that Zombi peas contain 14.5% protein when their roots are dried. Nevertheless, a more recent study conducted by Tripathi et al. (2020) to analyze the nutritional content of seven different Zombi peas accessions found that the protein content of their tubers ranged from 7.64% to 9.93%. This is remarkable because it was seven to nine times higher than the protein content found in sweet potato and cassava tubers (Tripathi et al., 2020). Although Zombi peas is not considered in this review, its rich nutritional contents particularly the tubers call for more research attention (Tripathi et al., 2020). The edible tubers of AYB and winged beans are still propagated at the subsistence level with no genetic improvement. The mechanism behind tuberization in AYB is yet to be understood.

**Figure 2:**
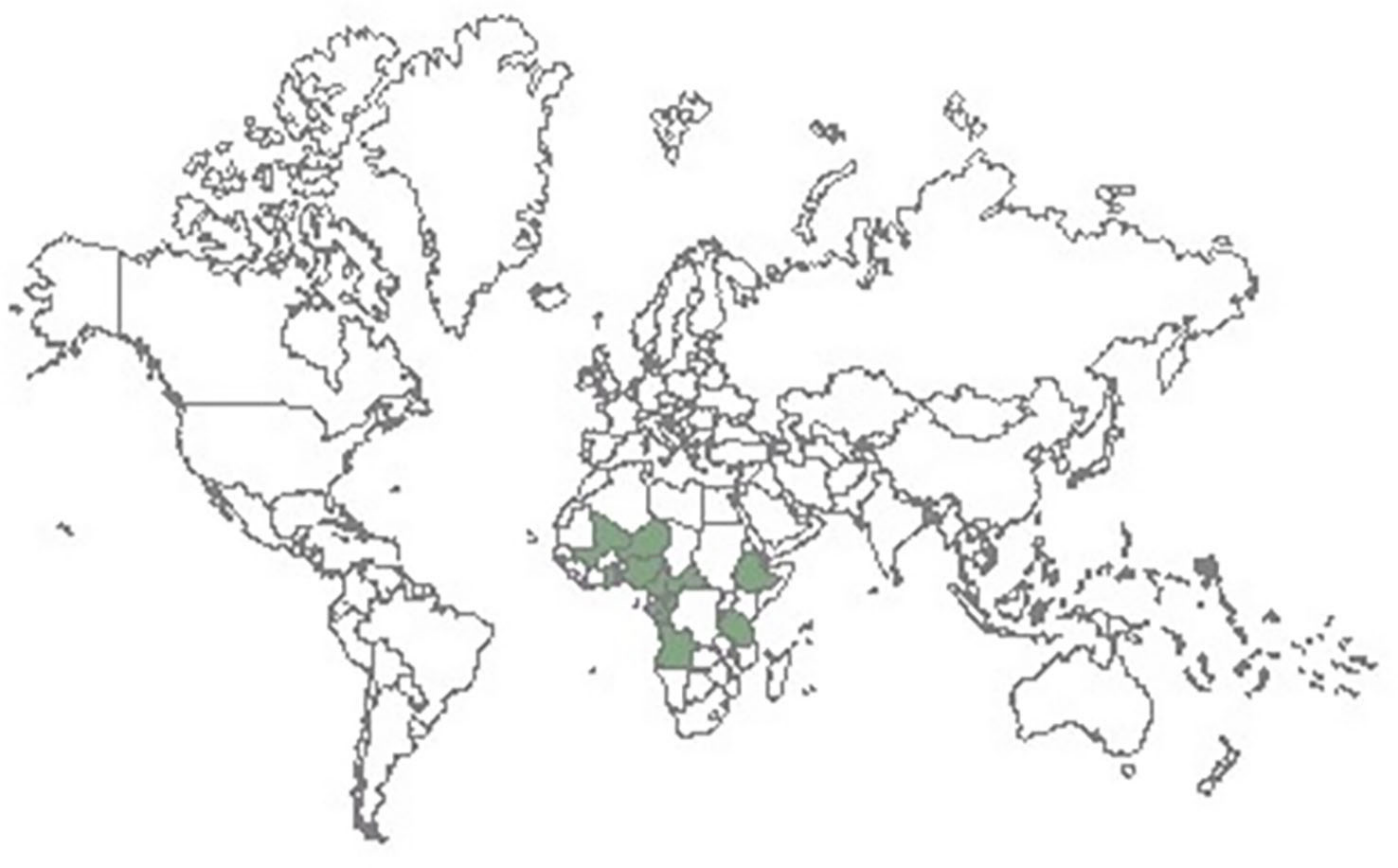
Center of the diversity of some underutilized legumes in Africa Source: http://www.zipcodezoo.com/Plants/S/Sphenostylis%5Fstenocarpa/Default.asp (February 19, 2023).

Bioactive compounds have been identified in NULs, but with little or no impact on the nutritional and food security in Africa. In plants, bioactives perform several functions, ranging from protection against herbivores and insect pest to attraction of pollinators during pollination and induction of essential functions (Chandrasekara and Josheph Kumar, 2016; Divekar et al., 2022). These bioactive compounds also exhibit pharmacological properties in humans and animals (Chandrasekara and Josheph Kumar, 2016) which forms a major part of this review. The bioactive components of the NULs are yet to be fully harnessed for improved health and well-being as many consumers in Africa are unaware of their nutritional and health benefits. In our previous review, we emphasized the need to integrate the NULs into food systems in sub-Saharan Africa (SSA) to cushion the negative effects of climate change, soil degradation, poverty, food insecurity, and malnourishment (Popoola et al., 2022b). This article attempts to present a broad review of some selected NULs, and their nutritional and functional properties. The selected NULs include Adzuki beans (*Vigna angularis*), African yam bean (*Sphenostylis stenocarpa*), Bambara groundnut (*Vigna subterranea*), Jack bean (*Canavalia ensiformis*), Kidney bean (*Phaseolus vulgaris*), Lima bean (*Phaseolus lunatus*), Marama bean (*Tylosema esculentum*), Mung bean, (*Vigna radiata*), Rice bean (*Vigna Umbellata*), and Winged bean (*Psophocarpus tetragonolobus*). In Africa, these NULs have been relegated to the status of “poor man’s food” with abysmally low level of cultivation, production, consumption, and utilization compared to the mainstream legumes. Consequently, the need to create awareness about their potential utility, health and nutritional benefits becomes imperative. Also, the relevance of the untapped bioactive compounds inherent in the seeds of these potential food and nutrition security crops are discussed. Furthermore, we highlight the prospects and current challenges associated with the utilization of these NULs and present strategies to facilitate their exploitation as not only sources of vital nutrients, but also integration for the development of cheap and accessible functional foods. The plant products and distribution of the selected underutilized legumes and center of diversity in Africa are presented in **Table 1** and **Figure 2**.

**Table 1:** Plant products and distribution of the selected underutilized legumes in Africa.

S/N	Common name	Botanical name	Seeds	Tuber	African Countries
1.	Adzuki beans	*Vigna angularis*	Present	Absent	DR Congo, Kenya, Angola, Zambia, Madagascar, Seychelles
2.	African yam bean	*Sphenostylis stenocarpa*	Present	Present	Nigeria, Ghana, Benin Republic, Cameroun, Togo, Niger, Kenya, Ethiopia, Mozambique, Tanzania.
3.	Bambara groundnut	*Vigna subterranea*	Present	Absent	Nigeria, Ghana, Niger, Mali, Côte d’Ivoire, Benin Republic, South Africa, Kenya.
4.	Jack bean	*Canavalia ensiformis*	Present	Absent	Western, Eastern, and Northern Africa
5.	Kidney bean	*Phaseolus vulgaris*	Present	Absent	Western, Eastern and Northern Africa
6.	Lima bean	*Phaseolus lunatus*	Present	Absent	Western, Eastern and Northern Africa
7.	Marama bean	*Tylosema esculentum*	Present	Present	Botswana, Namibia, Mozambique, Zambia, and northern South Africa
8.	Mung bean	*Vigna radiata*	Present	Absent	Nigeria, Liberia, Sierra Leone, Ghana, Côte d’Ivoire, and DR Congo
9.	Rice bean	*Vigna Umbellata*	Present	Absent	Egypt, Kenya, Tanzania, Burundi, Somalia, Rwanda
10.	Winged Bean	*Psophocarpus tetragonolobus*	Present	Present	Papua New Guinea Highland, northern Ghana, and Burma Nigeria, Togo, Benin

## 2 Neglected and underutilized legumes

The term “neglected” or “underutilized” alludes to a class of legumes that are climate-smart, adapted to marginal areas, indigenously propagated with fewer or no *ex-situ* collections, and have not given priority by policymakers. The term also refers to legumes that have received little research attention, possess local significance in production and consumption, traded regionally or internationally and are usually cultivated on a small scale by rural families for subsistence, particularly under adverse environment conditions (Cullis and Kunert, 2017; Yang et al., 2018; Rathi et al., 2021; Popoola et al., 2022b). While these crops have received relatively little research and funding, their potential is well recognized (Cullis and Kunert, 2017; Yang et al., 2018). The NULs exhibit an array of genetic diversity and exist as wild or cultivated species across different regions of the world (Agbolade et al., 2019). These crops are primarily grown by traditional farmers in SSA, Asia, and North America (Alvarado-Lopez et al., 2019). The NULs are marked by unique characteristics such as ethno-uses, seed sizes, growth habits, and fruiting patterns that distinguish them from the common pea. Furthermore, they are of agricultural importance owing to their capability to augment soils *via* symbiotic nitrogen fixation (Agbolade et al., 2019; Hunter et al., 2019; Popoola et al., 2022b). Underutilized legumes are a great source of essential nutrients such as dietary fiber, vitamins, polyunsaturated fatty acids (PUFAs), proteins with essential amino acids, minerals, and bioactive compounds. These legumes are therefore considered functional foods that can have positive effects on our health (Popoola et al., 2020; Rai et al., 2021).

African yam bean, Bambara groundnut, lablab bean, lima bean, and the winged bean are examples of some commonly cultivated underutilized leguminous species in SSA (Agbolade et al., 2019; Popoola et al., 2020). These legumes have the potential to drive sustainable agri-food systems in the region given their diversity, climate resilience, nutrient-dense nature, and cultural attachment to the regional food habits of the communities of origin (Paliwal et al., 2021). A recent analysis of the utilization and cultivation of lesser known legumes brought attention to the significant traits and potential prospects of some of the crops (Popoola et al., 2022b).

## 3 Nutritional properties of NULs

Nutritionally, the seeds of the selected NULs are rich in proteins, carbohydrates, minerals (calcium, manganese, phosphorus amongst others), and a wide range of vitamins (**Table 2**). Nutritional information on the tubers is scanty and has not been studied in detail compared to the seeds (Ojuederie and Balogun, 2019; Tripathi et al., 2020). Nevertheless, a few reports on AYB, Zombi pea, and winged beans indicate that the tubers are nutritionally rich with varied carbohydrates, proteins, ash, dietary fiber, minerals, and vitamin contents when compared to those of cassava, potato, and yam (Adegboyega et al., 2019; Konyeme et al., 2020; Ojuederie et al., 2020; Tripathi et al., 2020). The studies of Konyeme et al. (2020) emphasized the rich nutritional value of the tubers, while the investigation of Ojuederie et al. (2020) confirmed their safe consumption by humans and livestock. Globally, the demand for food legumes is ever-increasing as one of the essential nutritional and conventional food with health and pharmacological relevance (Tadele and Bartels, 2019). The nutritional contents of the seeds are diverse and have been widely reported and discussed by many researchers (Gagné-Bourque et al., 2016; Huang et al., 2018; Omotayo and Aremu, 2021; Popoola et al., 2022a). The nutritional profile of the seeds varies in different accessions of the same and different species. This can be exploited by breeders to enhance yield, taste and value chain for the food and confectionary industries. The amount of carbohydrates found in the selected underutilized legumes range from 18.90g in Marama bean (MB) to 70.48g in Kersting’s groundnut (KG) (**Table 2**). In legumes, carbohydrates usually contain resistant starch sugars such as stachyose, raffinose, and fructooligosaccharides. These sugars have the ability to improve the microbial environment in our gastrointestinal tract and promote gut metabolism (Johnson et al., 2020). Adding such components to food systems can greatly improve health and ensure nutritional quality. The proteins are of high quality and range from 7.80g in Lima beans (LB) to 29.60g in winged beans (WG) while others also contain a good quantity of protein. The amino acids found in these legumes are valuable in boosting the immune system, regulating metabolic processes, and enhancing glucose and fatty-acid metabolism (Tjahjadi et al., 1988; Semba et al., 2021; Ayilara et al., 2022). Numerous studies, including those conducted by Gohara et al. (2016), Nnamani et al. (2017), Baiyeri et al. (2018), Adegboyega et al. (2020), and Abberton et al. (2022), have documented the functions of various vitamins and minerals. Selected underutilized legumes (NULs) have been found to contain thiamine, niacin, riboflavin, vitamins A, B6, C, D, E, K, and pantothenic acid, according to these studies (**Table 2**). Underutilized legumes have been found to contain various mineral elements such as sodium (Na), calcium (Ca), copper (Cu), magnesium (Mg), manganese (Mn), phosphorus (Ph), potassium (K), and zinc (Zn) (Baiyeri et al., 2018; Nnamani et al., 2018; Adegboyega et al., 2019; Adedayo et al., 2021). The vitamins and minerals are required for optimal health and growth, improved memory, and blood circulation. Nevertheless, allergenicity, digestibility, and antinutritional factors (ANF) are major constraints to their functional utilization. However, various methods such as steaming, boiling, fermentation, irradiation, and high-pressure cooking have been found to overcome these challenges, as indicated in studies conducted by (Maphosa and Jideani, 2017; Bessada et al., 2019; Tan et al., 2020). Despite this, underutilized legumes, especially tubers, have not been fully utilized and their nutritional content has not been fully exploited (Ojuederie et al., 2020; Ayilara et al., 2022; Popoola et al., 2022a). The nutritional composition of the selected underutilized legumes’ raw, mature seeds, with values per 100g, is presented in **Table 2**.

**Table 2:** Nutritional composition of the selected underutilized legumes' raw, mature seeds, with values per 100g.

Nutrient contents	ADB	AYB	BG	JB	KB	LB	MB	MGB	RB	WB
Protein (g)	20.36	22.46	18.8	20.90	23.00	7.80	34.71	23.80	20.50	29.60
Carbohydrate (g)	62.26	53.68	61.30	60.61	70.48	20.90	18.9	61.00	51.31	41.70
Moisture (g)	13.07	9.53	2.10	2.19	Nr	Nr	2.80	9.80	Nr	Nr
Ash (g)	3.85	4.28	2.40	3.45	5.13	1.15	3.19	3.51	Nr	3.98
Fat (g)	0.45	3.59	6.20	1.59	1.38	0.38	40.06	1.22	0.60	16.30
Total Dietary Fibre	7.30	7.30	5.50	3.98	20.93	7.00	50.81	4.57	13.10	25.90
Water (g)	13.40	61.50	10.30	Nr	59.00	69.80	Nr	Nr	Nr	8.34
Energy (kcal/100 g)	334.80	333.67	367.00	338.00	386.39	115.00	544.57	344	318.00	409.0
Folates (B9) µg	0.62	0.10	0.25	0.40	1.11	83.00	0.14	0.62	Nr	45
Thiamine (B1) (mg/100g)	0.46	0.19	0.61	0.51	0.50	0.161	0.38	0.62	Nr	1.03
Niacin (B3) (mg/100g)	2.63	0.07	1.80	1.54	0.51	0.421	0.06	2.25	Nr	3.09
Riboflavin (B2) (mg/100g)	0.22	0.20	0.31	0.20	0.03	0.055	0.06	0.23	Na	0.45
Vitamin B6 (mg/100g)	0.35	0.10	0.44	0.51	4.67	0.161	9.21	0.38	Na	0.175
Vitamin A (mg/100g)	11.39	Nr	Nr	Nr	0.00	0.00	0.27	200	Na	Nr
Vitamin C (mg/100g)	0	12.97	0.27	8.10	0.55	0.00	0.00	4.80	Na	0
Vitamin D (mg/100)	0.00	0.00	3.42	0.00	0.00	0.00	132.9	0.00	Na	Nr
Vitamin E (mg/100g)	Nr	0.19	Nr	Nr	0.10	0.18	6.27	0.51	Na	Na
Vitamin K ((mg/100g)	Nr	Nr	0.001	Nr	14.90	2.00	0.22	9.00	Na	Na
Pantothenic acid (B5) (mg/100g)	1.47	Nr	1.80	Nr	0.40	0.422	Nr	1.91	Na	0.795
Sodium (mg/100g)	5.00	1.00	3.60	2.53	53.48	2.00	63.75	15.00	32.00	38
Calcium (mg/100g)	66.00	15.00	1.60	3.21	104.12	17.00	241.00	216	340.00	440
Copper (mg/100g)	1.09	0.29	0.09	0.43	0.40	0.235	1.04	1.27	1.12	2.88
Iron (mg/100g)	4.98	1.50	5.52	0.83	7.00	2.39	3.95	6.74	5.80	13.40
Magnesium (mg/100g)	127	69.00	7.58	1.95	118.95	43.00	274.50	204		179
Manganese (mg/100g)	1.73	3.35	0.26	0.35	0.80	0.516	1.85	1.23	0.68	3.72
Phosphorus (mg/100g)	381	99.00	32.50	1500	251.30	111.00	454.00	374	Na	451
Potassium (mg/100g)	1254	419.00	183.00	5.93	1517.36	508.00	895.00	1443	Na	977
Zinc (mg/100g)	5.04	0.78	0.27	2.90	2.38	0.95	6.20	1.88	3.39	4.48
Selenium (mg/100g)	3.10	150.00	Nr	Nr	2.10	4.50	0.08	8.2	Nr	8.20
Beta-carotin (µg)	Nr	7.00	0.47	Nr	Nr	0.00	Nr	68	Nr	Nr

ADB, Adzuki beans (Vigna angularis); AYB, African yam bean (Sphenostylis stenocarpa); BG, Bambara groundnut; (Vigna subterranea), JB, Jack bean (Canavalia ensiformis); KB, Kidney bean (Phaseolus vulgaris); LB, Lima bean (Phaseolus lunatus); MB, Marama bean (Tylosema esculentum); MGB, Mung bean (Vigna radiata); RB, Rice bean (Vigna Umbellata); WB, Winged Bean (Psophocarpus tetragonolobus); Nr, Not reported; Na, Not available.

Values adopted from the United States Department of Agriculture (USDA), Baiyeri et al. (2018), Charrondière et al. (2020) and Nnamani et al. (2018).

## 4 Functional properties and probiotics of underutilized legumes

### 4.1 Functional properties of underutilized legumes

Underutilized legumes possess noteworthy functional properties which are beneficial to food systems. Functional properties such as solubility, hydration, emulsification, foaming stability, gel-forming index, and pasting properties govern the utilization of legumes as protein-rich gluten-free food additives. The functional properties of these substances should be taken into account when formulating and processing food, to develop innovative food products (Bessada et al., 2019). Moreover, the functionality of legumes is affected by protein including its molecular size, structure, and charge distribution as well as non-protein molecules such as carbohydrates, lipids, and salts.

The functional properties of the underutilized legumes considered in this review are presented in **Table 3**. The hydration properties of flour are swelling power, solubility, water, and oil absorption capacities. These properties influence the structural, rheological, thermal, and sensory characteristics of foods. Swelling power is a measure of both intragranular and intergranular water present in flour/starch under excess water and high thermal conditions (Iuga and Mironeasa, 2020). According to recent research by (Palupi et al., 2021), lima bean flour has a swelling power of 6.88 g/g, a solubility of 18.68%, a water absorption capacity of 1.93 g/g, and an oil absorption capacity of 1.56 g/g when exposed to a temperature of 87°C. The African yam bean seed flour displayed good gelation properties, while protein solubility varied with pH, with high solubilities in acid and alkali (Oshodi et al., 1997) (**Table 2**). Ratnawati et al. (2019) revealed that at 95°C, mung bean and red bean flours had significantly higher swelling powers (10.5 and 10.1 g/g, respectively) than soybean flour (4.8 g/g). The research of Yadav et al. (2018) indicated that adzuki beans had a hydration capacity range of 0.05 to 0.12 g/seed, a swelling capacity of 0.04 to 0.15 mL/seed when the cooking time is reduced (48.67 to 74.33 min). Typically, flour with high swelling power elicits high gelatinization and paste properties which is vital for the structural and textural development of baked foods (Onyango, 2016). In addition, the solubility of flour or starch provides an index of the hydrophilic behavior of amylose molecules under high moisture and thermal conditions (Dudu et al., 2019). Flour solubility has an impact on the clarity of drinks as well as foam formation and stabilization. It also affects emulsification, gelation, and retrogradation which influences crumb grain formation, texture, sensory properties, and staling of baked foods. Furthermore, water and oil absorption capacities are the maximum amount of intragranular water or oil present in flour under excess moisture and ambient temperature conditions (Iuga and Mironeasa, 2020). Emulsification properties consist of emulsifying activity and stability which are modulated by the ratio of hydrophobic to hydrophilic amino acids present in the legume flour. Emulsifying activity is a measure of the ability of flour to form a stable emulsion (oil-water interaction) by protein dispersion in the presence of oil. On the other hand, stability measures the strength of the emulsion formed (Nawaz et al., 2021). Most of the underutilized legumes have been reported to show good functional properties of solubility, emulsification, oil absorption capacity, gelation and forming properties (Okezie and Bello, 1988; Onimawo et al., 1998; Barac et al., 2015; Arogundade et al., 2016; Mubaiwa et al., 2018; Diedericks et al., 2020; Yang et al., 2022).

**Table 3:** Functional properties of the selected underutilized legumes considered in this review.

Legume flour	Swelling power	Solubility	Water absorption capacity	Oil absorption capacity	Foam capacity	Emulsifying capacity	Gel Formation	Pasting Temperature	Peak Viscosity	Breakdown Viscosity	Setback Viscosity	Bulk Density	References
Adzuki bean	0.04 to 0.15 mL/seed	–	281.35%	252.27%	–	–	–	–	–	–	–	0.76 - 1.00 g/mL	(Yadav et al., 2018)
African yam bean	4.98 g/mL		2.01 g/mL	2.07 g/mL	5.01%	2.61 g/mL		83.55 °C	1108 BU	65.50 BU	768 BU		(Nonye and Chinasa, 2022)
	–	–	0.70%, 131.9% - 218.8%	1.48%	18.0%, 40.2%	56.67%, 50.7%	14.2%	–	–	–	–	0.63-0.87 g/mL	(Iwe et al., 2016; Obatolu et al., 2001)
Bambara groundnut			1.62-2.38 g/g	2.29-2.82 g/g		46%-55%		84-85.63 °C	892.50-1320.50 cP	98-157 cP	1414-1647 cP	0.58-0.71 g/mL	(Falade and Nwajei, 2015)
Jack bean		Temperature-dependent	Temperature-dependent	–				60 – 70°C					(Akinyemi et al., 2020)
Kidney bean			1.7- 2.7 g/g	1.4-1.7 g/g				79.2- 84.3°C	372-1015 cP	30- 67 cP	658- 1428 cP	1088-2385 cP	(Shevkani et al., 2022)
Lima bean	6.88 g/g	18.68 g/g	1.93 g/g	1.56 g/g					1172cP	83 cP	2377 cP	1288 cP	(Palupi et al., 2021).
Marama bean	–	6.6 g/g	1.50 g/g	2.7 g/g	31.1%	59.9%							(Maruatona et al., 2010)
Mung bean	10.50 g/g	18.80 g/g						77.9 °C	90.9 cP	40 cP	518.7 cP		(Ratnawati et al., 2019)
Rice bean	–	–	–	–	–	–	–	–	–	–		–	–
Winged bean	–	–	0.66 g/g	1.63g/g	13.67%	3.42 m2/g	–	–	–	–	–	–	

Foaming properties include foaming capacity and stability. Foaming capacity is the ability of protein or flour to add air when whipped, and foam stability is the capacity to stabilize foams (volume) over time (≤30 min) (Bessada et al., 2019). Iwe et al. (2016) revealed that African yam bean flour has a foaming capacity and stability of 18% and 92.6%, respectively. Pasting properties, mainly pasting temperature, peak, breakdown, and setback viscosities provide insights into swelling capacity, structural stability, and amylose retrogradation tendency of flour under combined high mechanical shearing and hydrothermal conditions. This is typically carried out in a rapid viscosity analyzer or a Brabender Visco-Amylo-Graph. These properties are critical to the functionality of flour in food and industrial systems. Pasting temperature is the thermal energy required to destroy flour granule structures leading to the onset of paste development. Associations between flour composition, pasting, viscosity, and bulk density of some underutilized legumes have been investigated (Du et al., 2014). Furthermore, some studies on legume flour highlighted the relationship of flour microstructure with pasting even though such studies are scanty for the underutilized legumes (Shevkani et al., 2021). The pasting properties of the processed lima bean flour showed a peak of 1172 cP, a breakdown of 83 cP, final of 2377 cP, and a setback viscosity of 1288 cp (Palupi et al., 2021). Ratnawati et al. (2019) revealed that the pasting temperature of red kidney bean flour (81.7 °C) was higher than that of mungbean flour (77.9 °C). Flours with low breakdown viscosity can serve as structuring agents in food where a minimal structural breakdown is required. Setback viscosity is the recovery of viscosity during the cooling of flour after being subjected to combined high mechanical shearing and hydrothermal conditions (Cornejo-Ramírez et al., 2018). It is an index of the retrogradation ability of flour, which is an important prerequisite for staling activity during product storage. Flours with low setback viscosity may be utilized in delaying retrogradation activity in cooked infant formulas, breakfast foods, and pasta products as well as prevent staling of baked products.

Bulk density is an index of the structural integrity of granules that relates to the packaging and raw material handling of flour (Adedayo et al., 2021). Iwe et al. (2016) revealed that AYB has a loose bulk density of 0.63 g/mL, repacked bulk density of 0.87g/mL, water absorption of 0.70%, and oil absorption of 1.48%, all of which are comparable to that of cowpea and rice. Bulk density in adzuki beans range 0.76 to 1.00 g/mL (Yadav et al., 2018). Nwajagu et al. (2021) showed that *M. flagellipes* seed flour has a bulk density of 0.8 mg/100g. Okechukwu-Ezike et al. (2020) revealed that black-eyed beans and black beans have higher bulk densities (0.6 g/cm3) than brown beans (0.4 g/cm3). Sattar et al. (2017) showed that black gram (*Vigna mungo*) flour, green gram (*Vigna radiata*) flour, and lentil (*Lens culinaris*) flour have bulk densities of 0.5 g/cm3, 0.5 g/cm3, and 0.6 g/cm3, respectively. Flours with high bulk density are suitable as thickeners, while those with low bulk density can be in complementary food formulas. The functional properties of the underutilized legumes considered in this review are presented in **Table 3**.

### 4.2 Probiotics and prebiotics potential of underutilized legumes

According to The International Scientific Association for Probiotics and Prebiotics (ISAPP), “Probiotics are live microorganisms, which when administered in adequate amounts, confer a health benefit on the host” (Marco et al., 2021). Moreover, prebiotics are “substrates that are selectively utilized by host microorganisms conferring a health benefit” (Sanders et al., 2019). In time past, the health benefits of probiotics were realized from the consumption of milk, soybean, and other dairy products. However, the problem of short shelf-life, allergenic milk proteins, high cholesterol content, lactose intolerance, consumer inclination towards veganism, and economic considerations for developing countries, have compelled the exploration of non-dairy alternatives with good nutritional profile and health-promoting factors (Panghal et al., 2018; Chaturvedi and Chakraborty, 2021; Chaturvedi and Chakraborty, 2022). In the last decade, the non-dairy food products market has received positive acceptance and is projected to reach an estimated 26 billion USD by the year 2025. The underutilized legumes hold an exceptional capacity to be utilized as probiotic carriers (Rasika et al., 2021; Chaturvedi and Chakraborty, 2022). These legumes constitute appropriate matrices for the production of non-dairy alternatives like plant-based beverages due to the presence of natural prebiotics including resistant starch, oligosaccharides, isoflavones, and polyphenols. These prebiotics exert a wide range of physiological functions such as immune system modulation, metabolic regulation, and anti-inflammatory and anti-cancer properties, and therefore offer enormous potential for the development of symbiotic foods (a blend of prebiotics and probiotics) using lactic acid bacteria (LAB) (Chaturvedi and Chakraborty, 2022; Cichońska and Ziarno, 2022).

Generally, research findings have shown that underutilized legumes such as adzuki beans, African yam bean, Bambara groundnut, and mung beans exhibit a low glycemic index due to their high resistant starch and amylose contents and have been shown to reduce the risks of high blood pressure and type-2 diabetes (Barac et al. 2015; Johnson et al., 2020; Adedayo et al., 2021; Johnson et al., 2022). Adeoye et al. (2021) reported the probiotic nutraceutical potential of Bambara groundnut. In the study, *Lactobacillus delbrueckii*, *L. casei* and *L. brevis* were the preponderant LAB found in isolates of fermented Bambara groundnut. The authors further demonstrated the *in vitro* antagonistic properties of the LAB isolates against pathogenic namely *Salmonella sp, Escherichia coli, Staphylococcus sp, Shigella sp and Pseudomonas* sp. Very recently, Chaturvedi and Chakraborty (2022) conducted a study to evaluate the prebiotic characteristics of synbiotic drinks made from legumes, specifically red kidney beans and green mung beans. The results indicated that these drinks had a considerable impact on promoting the growth of the probiotic Lactobacillus casei ATCC 335, while simultaneously hindering the colonization of the enteric pathogen *Escherichia coli*. The study established that the formulated beverage showed prebiotic and probiotic potentials that could serve as a veritable alternative to dairy symbiotic beverages. Thus, the functional and probiotic properties elicited by the above-mentioned underutilized legumes serve as a point of reference for their suitability and/or exploitation in food, industrial and pharmaceutical systems. However, more research is required in this regard to provide more insight into the probiotic and prebiotic potentials of underutilized legumes.

## 5 Bioactive components of underutilized legumes

**Figure 3:**
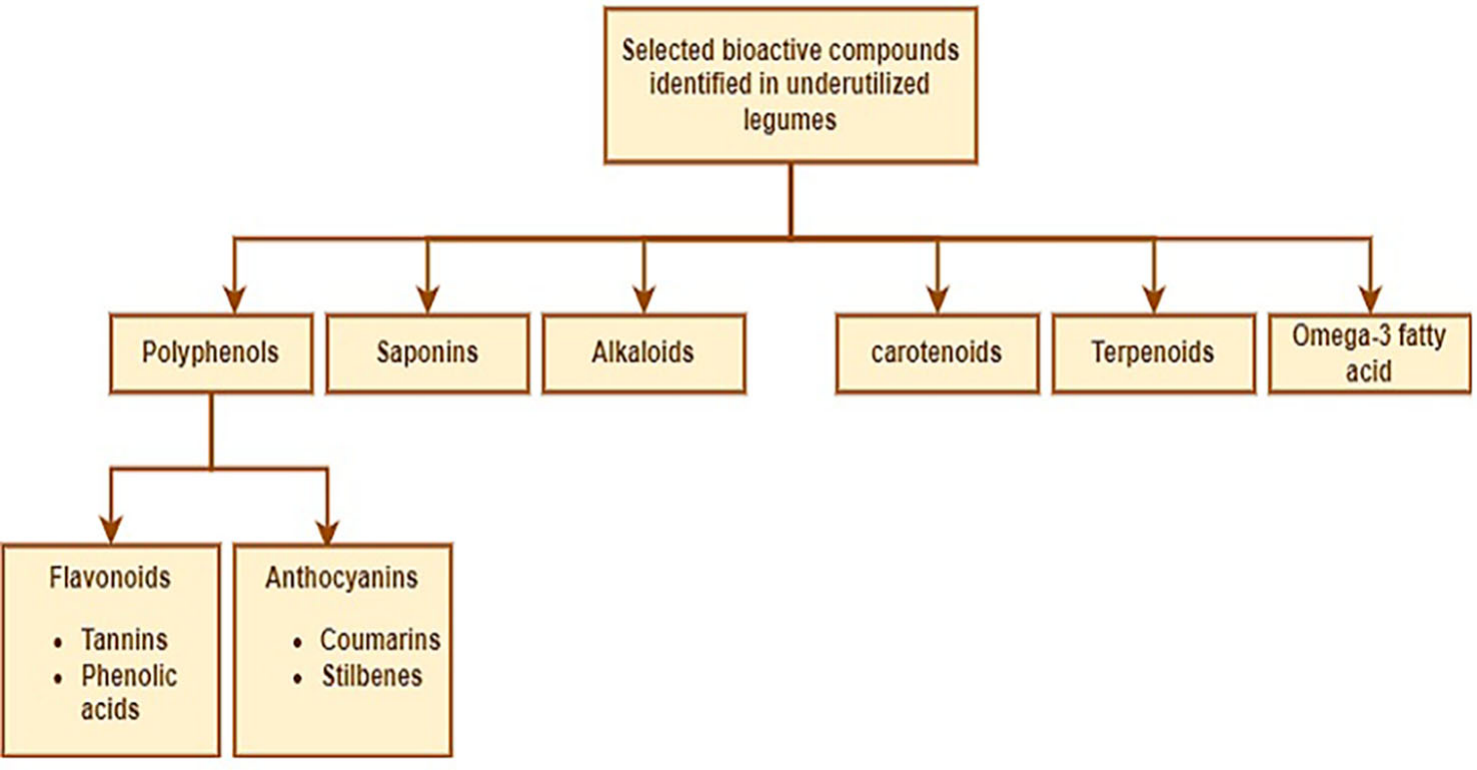
Selected bioactive compounds in underutilized legumes.

Bioactives are compounds that when ingested by humans/animals have some physiological contributions that could enhance healthy living and support a decrease in the occurrence of illness (James et al., 2020; Cui et al., 2021). Commonly, legumes including the underutilized ones are rich in polyphenols, alkaloids, saponins, carotenoids, terpenoids, omega-3 fatty acids, flavonoids, and anthocyanins amongst others (**Figure 3**). These substances have varying degrees of abilities to act as antioxidants, antimicrobials, anticancer agents, anti-tumor agents, anti-inflammatory agents, and neuroprotective agents (Zheng et al., 2019; Shevkani et al., 2022; Popoola et al., 2022a). Furthermore, it has been suggested that polyphenols, alkaloids, and saponins play a vital role in protecting the plant from herbivores and pathogens by serving as defense mechanisms. They also act as signaling molecules between the plant and its biotic environment (Divekar et al., 2022).

Several authors such as Oboh et al. (2015); Ajibola and Olapade (2016); Soetan (2017); Soetan and Atanda (2018); Soetan and Adeola (2018); Soetan et al. (2018); Adegboyega et al. (2019); Mayes et al. (2019); Ojuederie and Balogun (2019); Adegboyega et al. (2020); Adegboyega et al. (2021); Ikhajiagbe et al. (2021); Ojuederie et al. (2021) and Popoola et al. (2022a) have attempted to unravel the bioactive ingredients available in underutilized legume crops such as African yam bean, Bambara groundnut, Kersting’s groundnut, and winged bean which can be exploited as nutraceuticals. In general, the seeds contain bioactive compounds that can improve human health and provide several benefits, such as aiding digestion, promoting weight loss, and reducing the risk of heart diseases and type 2 diabetes (Alcázar-Valle et al., 2020; Bhadkaria et al., 2021). In addition to the dietary fiber, polyphenols, and natural antioxidants embedded in the seeds are of vital benefits to defend against free radicals (Amarowicz and Pegg, 2008; Xu et al., 2017). These components are urgently needed to be exploited for the benefit of man and animals particularly in the management of degenerative infections (Silva et al., 2007; Singh et al., 2017). The promising nature of these compounds (**Figure 3**) has afforded researchers to look into the possibilities of developing food-based therapy for disease management but more actions are still required in this direction (James et al., 2020).

Polyphenols and their derivatives such as flavonoids, anthocyanins, tannins, and tocopherol are among the essential bioactive compounds found in underutilized legumes. In a comparative study of antioxidants produced by Bambara groundnut (BG) using methanolic extract, Salawu (2016) identified the presence of several polyphenols in the raw and cooked BG seeds after quantification using HPLC-DAD. A recent study identified catechin, epicatechins, rutin, quercetin, iso-quercetin, kaempferol, luteolin, gallic acid, chlorogenic acid, caffeic acid, and ellagic acid as polyphenols found in BG (Okafor et al., 2022). Likewise, Harris et al. (2018) compared the level of flavonoids in red and brown BG hulls and observed that the brown hull had the highest amount of rutin (24.46 ± 0,23 mg g^-1^) and myricetin (1.80 ± 0.77 mg g^-1^) while the phytochemicals chlorogenic acid and ellagic acids which are tannins, had their highest concentrations in red BG hulls (0.12 ± 0.19 mg g^-1^) and brown BG (0.11 ± 0.08 mg g^-1^) respectively (Harris et al., 2018). Their findings revealed that the best source of flavonoids and tannins were found in the brown and red hulls rather than in the whole or dehulled BG seeds.

Apart from producing antioxidants, polyphenols are also known to possess anti-microbial, anti-viral, anti-inflammatory, anti-allergic as well as anti-mutagenic effects, scavenging free radicals that cause cell degeneration and death. The anti-cancer properties of some of these polyphenols have been previously tested and confirmed. For instance, ellagic acid, quercetin, catechin, and phenolic acid prevented several kinds of cancer that affect the skin, stomach, duodenum, mouth, colon, liver, lung, and mammary glands (Yang et al., 2001; Okafor et al., 2022). Ade-Omowaye et al. (2015) and Soetan et al. (2018) independently confirmed the presence of antioxidant-related phytochemicals: phenolics and flavonoids in AYB. Out of nine underutilized legumes studied by Ade-Omowaye et al. (2015), AYB had very high total polyphenolic contents of 293.23 mg 100 g^-1^ and 288.68 mg 100 g^-1^ with higher antioxidant activities, (1.00 mmoleTE 100 g^-1^ and 0.67 mmolTE 100 g^-1^), respectively, including a variety of BG (0.88 mmolTE 100 g^-1^). Akinyemi et al. (2020) confirmed the presence of flavonoids, tannins, alkaloids, saponins, and cardiac glycosides in Jack beans. According to several studies (Sowndhararajan et al., 2011; Solomon et al., 2018; Akinyemi et al., 2020), Jack beans have high levels of antioxidants that have been shown to possess numerous health benefits such as reducing the risk of type 2 diabetes, cancer, and inflammation, improving lipid metabolism, lowering bad cholesterol levels, preventing metabolic syndrome, and reducing the incidence of cardiovascular diseases. Legume seeds contain significant amounts of antioxidants due to their high phenolic, flavonoid, and anthocyanin contents. Consuming products made from these legumes may help prevent and manage various chronic and degenerative diseases, as well as address protein-calorie malnutrition (Foyer et al., 2016; Tsamo et al., 2020; Popoola et al., 2022a). Studies by Adefegha and Oboh (2012); Kennedy (2014); Adefegha et al. (2017); Gupta and Prakash (2019), and Adefegha (2018) have confirmed that these bioactive components of NULs could play significant roles in increasing the immune level and support prevention of common diseases such as malnutrition (severe and acute particularly in infants), sexual enhancers, obesity, diabetes, heart-related diseases amongst others.

In terms of potentials as nutraceuticals, African yam bean (*Sphenostylis stenocarpa*), and Horse gram (*Macrotyloma uniflorum*) are legumes worthy of note, due to their anti-diabetic property, anti-urolithiasis effect, and role in the prevention and management of cardiovascular diseases, kidney stones, gastritis, pile, and urinary tract disease (Sharma et al., 2019; Vijayakumar, 2021). Extracts from mung bean, adzuki bean, black bean, rice bean, and lima bean have been documented to exert hepato-protective effects due to the presence of antioxidant and anti-inflammatory compounds (Vijayakumar, 2021). The bioactive components and health benefits of the selected NULs are presented in **Table 4** and **Figure 3**.

**Table 4:** Bioactive components of the selected neglected and underutilized legumes.

S/N	NULs Food Sources	Phytochemicals/Bioactive Contents	Health Benefits	References
1.	Adzuki beans	Phenol (tocopherols) and Flavonoids	Antioxidant activities, anti-atherogenic, anti-thrombogenic, and hypochloremia effects	(Gohara et al., 2016; Luo et al., 2016; Johnson et al., 2022)
2.	African yam bean	Phenols and flavonoids Resistant starch, slowly digestible starch (SDS), and non-starch polysaccharides	Antioxidant activities as nutraceuticals Stabilizes glucose metabolism and insulin levels improves mental performance and modulates appetite	(Huang et al., 2018; Soetan et al., 2018; George et al., 2020; Lin Tan et al., 2020)
3.	Bambara groundnut,	Tannins and flavonoids	Neuroprotective, cardioprotective, antitumor, and antioxidant properties	(Lin Tan et al., 2020; Adedayo et al., 2021)
4.	Jack bean	Polyphenols: flavonoids, tannins, alkaloids, saponins, and cardiac glycosides	Antioxidant with anti-diabetic, anti-cancer, and anti-inflammatory properties. Improves lipid metabolism, prevents metabolic syndrome, lowers bad cholesterol levels, and reduces cardiovascular diseases incidence and cancer risk	(Akpapunam and Sefa-Dedeh, 1997; Sowndhararajan et al., 2011; Akinyemi et al., 2020)
		Haemagglutinins	Anticancer and Immunostimulant activities	(Carbonaro et al., 2015)
5.	Kidney bean	Phenolic (tocopherol, total phenolics, total flavonoids and antioxidant activities	Promotes weight loss, anti-cholesterol, and anti-diabetic, and hepatoprotective properties.	(Kan et al., 2017; Idoko et al., 2020)
6.	Lima bean	Polyphenols, Flavonoids and Tannins	Anti-diabetic, antifungal, antiproliferative properties, hepaprotective activity, antioxidant effects, trypsin, hypocholesterolemia activities	(Agostini-Costa et al., 2014; Drago et al., 2016)
7.	Marama bean	Oleic acid Stearic acid Palmitic acid Polyphenol	Enhances glucose homeostasis and anti-inflammatory activity. Reduces blood pressure and atherosclerosis risk, improves heart function Hypolipidemic and anti-inflammatory properties associated with the prevention of cardiovascular disease, metabolic syndrome, and diabetes-related insulin resistance. Antioxidant, anti-bacterial, anti-fungal, anti-inflammatory, antihyperglycemic and pro-apoptotic properties; protects against free radical-induced erythrocyte hemolysis; represses rotavirus-induced inflammation	(Omotayo and Aremu, 2021; Omotayo and Aremu, 2019)
8.	Mung bean	Tannins and phytic acid	Attenuates blood glucose level and insulin responses to plasma cholesterol and starchy foods reduces cancer risks	(Gemede and Ratta, 2014; Ganesan and Xu, 2018)
9.	Rice bean	Phenols and Flavonoids Phenolic acids (*p*-coumaric acid, ferulic acid, and sinapic acid) Flavonoids (catechin, epicatechin, vitexin, isovitexin and quercetin)	Antidiabetic properties including α-glucosidase inhibition and advanced glycation end-product formation inhibitory activities.	(Yao et al., 2012; Bhagyawant et al., 2019; Kaur et al., 2021)
10.	Winged Bean	Polyphenol	Anticarcinogenic, antioxidant, anti-inflammatory, antitumoral, antimicrobial, antimutagenic, anti-ischemic and anti-allergic properties	(Mohanty et al., 2013; Bassal et al., 2020)
		Lectin	Antiploliferative activity	(Kortt, 1984)

Bioactive proteins and peptides are abundant in legume seeds (Kortt, 1984; Mojica and González de Meija, 2015; Makeri et al., 2016; Hussein et al., 2020). A notable bioactive protein in legume seeds is a lectin. Lectins possess anticancer and immunostimulatory activities. Lectins also help to reduce the risk of cardiovascular diseases in obsessed individuals (Roy et al., 2010; Carbonaro and Nucara, 2022).

Haemagglutinins from Jack bean possess anticancer and immunostimulatory properties (Carbonaro and Nucara, 2022). Jack bean produces a well-known lectin called Concanavalin A (Con. A) which has an extremely high anti-hepatoma activity arising from its resistance and structural stability to *in vitro* proteolysis and denaturation (Carbonaro et al., 2015; Huldani et al., 2022). The high level of lectins in winged bean has also been shown to have antiproliferative activity on human cancer cell lines. Two lectins (B2 and B3) were identified in winged bean which exhibited the same amino-terminal sequences and the sequence of lectin B3 to residue 40 reflected extensive homology with other legume lectins such as soybean lectin (Kortt, 1984).

Saponins are often regarded as antinutritional factors (ANTs) in grain legumes as they inhibit active transport and simultaneously increase the general permeability of enterocyte barrier (Bennetau-Pelissero, 2018). Thus, saponins increase the permeability of the small intestinal mucosal cells, facilitating the uptake of substances to which the gut would normally be impermeable. It also reduces the bioavailability of nutrients and decreases enzyme activity, resulting in an inhibition of growth. Notwithstanding, saponins have some positive health benefits as they contain a triterpenoid aglycone (sapogenin) linked to one or more oligosaccharide groups with the ability to absorb free radicals and activate antioxidant enzymes (Hai et al., 2021). Saponins in legume seeds contain two major components soya-saponin I (approximately 630 to 900 mg/kg) and dehydrosoyasaponin I (approximately 650 to 1300 mg/kg). The hilum portion of legume seeds has been identified as having the highest saponin content compared to the cotyledons (Hai et al., 2021). Research findings reveal that Japanese and Chinese populations have a lower risk for breast, colon, corpus uterine, and prostate cancers due to their high intakes of legumes and legume products, which are good sources of saponins (Messina et al., 1994; Shi et al., 2004). Thus, they tend to have a longer life span than Africans (Lu et al., 2017). Terpenoids are a sub-group of triterpenoids and have been implicated to reduce bad cholesterol level and possess anti-cancer and antimicrobial properties (Marrelli et al., 2016). To our knowledge, little or no research has been done on the benefits of saponins and terpenoids derived from African underutilized legumes. This is an aspect of research that should be promoted to enhance the livelihood of the African population and would aid in the attainment of the Sustainable Development Goal of the United Nation on better health and well-being.

Alkaloids just like saponins are considered ANTs, and have been reported in a few underutilized legumes but not in detail (Konyeme et al., 2020; Popoola et al., 2022a). Alkaloids are naturally essential as defense agents which make up approximately 20% of the known secondary metabolites available in plants (Kaur and Arora, 2015). Therapeutically, alkaloids are particularly well-known as antioxidants, anti-inflammatories, anesthetics, and cardioprotective agents (Kurek, 2019; Heinrich et al., 2021). The presence of alkaloids in some underutilized legumes (winged bean, AYB, and Kersting’s groundnut) suggests their potential application as anti-cancer, anti-inflammatory, antimicrobial, and analgesic agents amongst others (Kaur and Arora, 2015; Popoola et al., 2022a). Jack bean and AYB have been found to exhibit higher contents of alkaloids (0.645g/100g) and (22.195-183g\100g) (Konyeme et al., 2020). More investigations are needed on underutilized alkaloids, especially about their contents and variability as regards quinolizidine (QA) and pyrrolidone (PA).

Carotenoids such as β-carotene, lutein, and cryptoxanthin have been detected in most legumes though much lower compared to that of fruits and vegetables (Tee et al., 1995). Carotenoids are widely distributed in legumes and have been reported to exhibit health-promoting benefits such as antioxidants, better visual function, and reduction of cardiovascular diseases (Voutilainen et al., 2006; Ku et al., 2020; Maoka, 2020). In a study to evaluate bioactive components of selected underutilized legumes indigenous to Nigeria, James et al. (2020) found out that, fermentation and germination reduced carotenoid, anthocyanin, tannin, and flavonoid contents of the legumes.

Lipid profiles of underutilized legumes has been fairly documented but their effects on blood lipid levels are limited (Zhang et al., 2010; Adebowale et al., 2011). However, research has linked the consumption of these diets to a decreased risk of heart disease and obesity, according to (Hossain et al., 2016). Furthermore, Yao et al. (2015) discovered that the Ci12 landrace of Bambara groundnut from Côte d’Ivoire contained a high concentration of n-6 fatty acids, which are classified as polyunsaturated fatty acids (PUFAs) and include Omega-6 linoleic acid (C18:2, ώ-6) and Omega-3 alpha-linoleic acid (C18:3, ώ-3). These acids cannot be produced by the body and must be obtained through diet. Studies have also shown that consuming diets rich in Omega-6 fatty acids can reduce the incidence of cardiovascular disease and obesity, as noted by (Patterson et al., 2012; Djuricic and Calder, 2021). Oleic, stearic and palmitic acids have been recorded for Maraba bean (Omotayo and Aremu, 2021). The fatty acids components such as palmitic, palmitoleic, oleic, arachidonic, eicosapentaenoic, docosapentaenoic, lignoceric, docosahexaenoic and nervonic acids have not been studied extensively in African underutilized legumes. Further studies are required to unravel the PUFAs available in these lesser-known legumes.

## 6 Antimicrobial properties of underutilized legumes

Infections resulting from microbial sources are a great source of threat to plants, animals, and human health, which have necessitated the use of effective, safe, and sustainable biocontrol methods. This is particularly important due to the resistance of microbes to antibiotics and other control mechanisms as well as the search for novel antimicrobial agents (Udeh et al., 2020). Underutilized legumes are embedded with inherent antimicrobial abilities through the presence of different phytochemicals which include phytate, tannins, anthocyanin, flavonoids, etc. (Ayilara et al., 2022). These phytochemicals have been reported to be capable of controlling both gram-positive and gram-negative pathogenic bacteria (Ramatsetse et al., 2023). For instance, Bambara groundnut has been reported to inhibit the growth of different human pathogenic organisms which include *Klebsiella pneumonia, Escherichia coli, Bacillus aureus, Pseudomonas aeruginosa, Candida albicans, Klebsiella aerogenes, Aspergillus niger* and *Staphylococcus aureus* (Klompong and Benjakul, 2015; Wanyama et al., 2017; Oyeyinka et al., 2021) (**Table 5**). Antimicrobial properties of other selected underutilized legumes are shown in **Table 5**. The mechanism of action of the underutilized legumes as antimicrobial agents includes disruption of the microbial activity, chelation of crucial micro mineral elements (zinc and iron), suppression of the cell surface microbial enzymes, hydrophobic and electrostatic interaction with the cell membrane and cell wall (leading to the production of large pores and consequently its disintegration), induction of morphological changes in bacteria cells, increase in the permeability of cell wall which results to cell lysis and death, penetration of the cytoplasmic membrane, reduced intracellular ATP concentration and the prevention of spore germination and mycelial growth in fungi (Sitohy et al., 2013; Lopes and Brandelli, 2018; Udeh et al., 2020; Jia et al., 2021). On antifungal potentials, an array of proteins/peptides from mungbean, kidney bean, African yam bean, lima beans, brown kidney, winged beans), have elicited antifungal effect against plant and human pathogens including *Fusarium oxysporum* and *Coprinus comatus, Verticillium dahlia, Botrytis cinerea, Setosphaeria turcica, Rhizoctonia solani, Mycosphaerella arachidicola, Helminthosporium maydis, Candida albicans, Gibberalla sanbinetti, Sclerotinia sclerotiorum*, etc. (Mani-López et al., 2021). More research focus is required on arrays of antimicrobial properties of underutilized legumes of Africa which will possibly lead to cheaper means of drug discovery and good health care in Africa.

**Table 5:** Antimicrobial properties of underutilized legumes.

Plant	Botanical name	Extractant	Part used	Microbe or disease control	References
Horse gram	*Dolichos biflorus*	Water	Seeds	*Escherichia coli, Pseudomonas aeruginosa, Bacillus subtilis* and *Staphylococcus aureus*	(Basu et al., 2017)
Mung bean Bengal gram	*Vigna radiata* *Cicer arietinum*	Water	Hull	*Bacillus cereus*	(Kanatt et al., 2011)
Lablab bean	*Lablab purpureus*	Peptide	Seeds	*Bacillus cereus*	(Bai-Ngew et al., 2021)
Bambara groundnut	*Vigna* *subterranea*	Water	Hull, seeds	*Klebsiella pneumoniae, Aspergillus niger, Pseudomonas aeruginosa, Candida albicans Staphylococcus aureus*, *Escherichia coli* and *Bacillus cereus*	(Udeh et al., 2020)
Adzuki bean	*Vigna angularis*	Ethanol	Seed coat	*E. coli* and *Staph aureus*	(Jia et al., 2021)
Kidney bean	*Phaseolus vulgaris*	Methanol	Seeds	Multidrug-resistant *Enterobacterales*	(Ebrahim et al., 2022)
Marama bean	*T. esculentum*	Water	Testae	*Campylobacter jejuni*, *Staphylococcus* sp., *Escherichia coli*, *Shigella* sp., *Yersinia* sp., MRSA, and *Salmonella* sp.	(Chingwaru et al., 2015)
Mung bean	*Vigna radiata*	Ethanol	Seed Flour	*L. monocytogens*, *C. jejuni, S.aureus, E.coli, B. subtilis* and *Pseudomonas aeruginosa*	(Keawpeng et al., 2022)
Winged bean	*Psophocarpus tetragonolobus*	Methanol	Leaves	*Pseudomonas aeruginosa*	(Latha et al., 2007)

The species of legumes, the concentration of the extract, and the type of extractant (solvent) used are essential factors that affect the activity of underutilized legumes as antimicrobial agents (Shelke et al., 2022). Kaundal et al. (2019) reported that when different solvents (dichloromethane, 1-butanol, water and ethyl acetate) were used to assess the antimicrobial properties of Horse gram against human pathogenic organisms (*Bacillus* sp.*, E.coli, Shigella* sp.*, Staphylococcus* sp. *and Salmonella* sp.), ethyl acetate and dichloromethane extracts revealed antibacterial activities while the aqueous and 1-butanol extract showed no antibacterial properties (Kaundal et al., 2019). Hence, it is essential to carry out further research on different underutilized legumes to unravel the best extractant that can be used to extract the active ingredients in different NULs to promote their potential in the discovery of new drugs.

In addition, different plant parts are used in the production of plant extracts, these include the pods, seeds, flowers, hall, root, stem, tuber, and leaf, where different types and different forms of phytochemicals can be found (Henciya et al., 2017; Zhong et al., 2022). More research should be carried out on underutilized legumes to unravel the different parts of each NULs that can give a maximum recovery and variety of antimicrobial active compounds.

## 7 Prospects in harnessing the benefits of underutilized legumes

Too much reliance on a few staple crops to meet the food and nutritional needs of man is a potential threat to the global fight against food insecurity and to ensure that the zero hunger sustainable development goals (SDGs) are achieved by 2030. Traditional or indigenous food crops in Africa have major roles to play in realizing the SDGs 2 and 3 of the United Nations if given the utmost attention and necessary improvement for human consumption. The African populace needs to be sensitized to the benefits derived from her indigenous legumes. Furthermore, researchers in Africa must embark on collaborative research and give priority to these legumes in crop improvement programs using a holistic approach. Cellular oxidative stress has been implicated in the development of chronic diseases such as cardiovascular disease, cancer, arthritis, diabetes, and degenerative diseases in humans. Nevertheless, antioxidants in foods regulate and reduce oxidative destruction by inhibiting oxidation caused by reactive oxygen species (ROS), and improve the shelf-life and quality of these foods (Ames et al., 1993; Altemimi et al., 2017). The bioactive components of legume seeds possess antioxidant activity which could mitigate the effects of oxidative stress. However, these bioactive forms a small percentage of the nutritional components of legume seeds.

**Figure 4:**
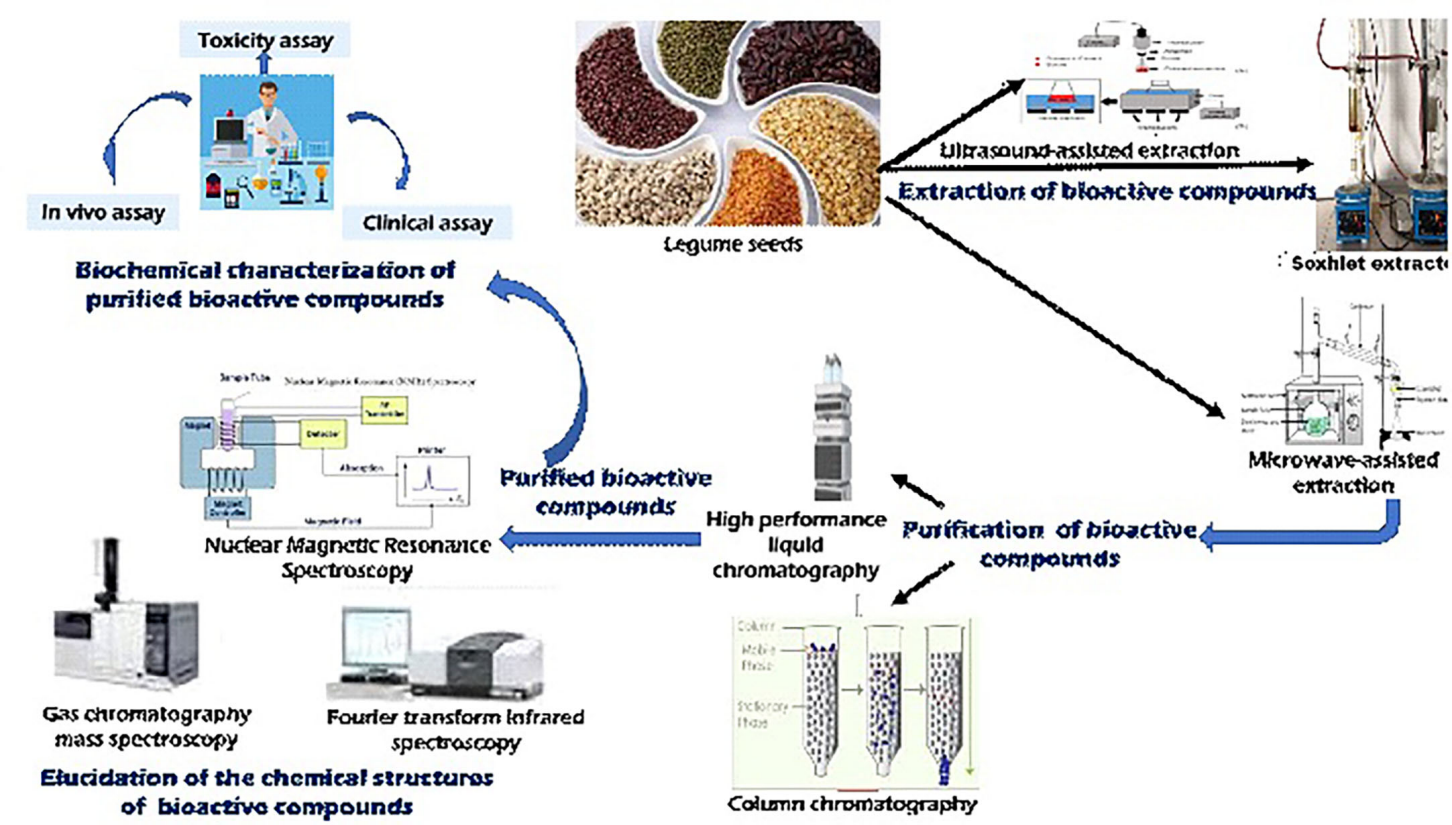
Extraction, characterization, and purification methods for legume seed bioactives.

Singh et al. (2021) revealed that bioactive compounds are concentrated in different parts of the seeds of legumes. For instance, phenolic compounds such as flavonoids and dietary fibers occur in the seed coat while non-flavonoids such as oligosaccharides and dietary fiber occur in the cotyledons (Singh et al., 2021). Including dietary fibers from legume seeds and cotyledons in one’s diet has several positive effects on human health. These fibers can assist with digestion in the gastrointestinal tract by increasing water-holding capacity, viscosity, bulk, fermentability, and the ability to bind bile acids, as noted by Stosh and Yada (2010). In addition, it is known to reduce serum cholesterol in hypercholesterolemic people and postprandial glycemia. The dietary fiber in the seed testa has been reported to be significantly higher than the quantity in the cotyledons (Sergio et al., 2020). These bioactive compounds could also be used to design functional food products. Unfortunately, most of the bioactive components of the seeds of underutilized grain legumes are unknown. Therefore, there is a need for these bioactives present in Africa’s indigenous legumes to be extracted, purified, and characterized using biochemical approaches with the chemical structures elucidated with the aid of high-tech equipment (**Figure 4**). To extract the bioactive compounds from these NULs, modern extraction techniques can be employed for maximum efficiency. Once extracted, these components are further purified using chromatographic methods, such as high performance liquid chromatography (HPLC) or column chromatography, before being profiled using analytical techniques such as nuclear magnetic resonance (NMR), gas-chromatography-mass spectroscopy (GC-MS), GC-time of flight-MS (GC-TOF-MS) which improves resolved peaks, LC-MS, or Fourier Transform Infrared spectroscopy (FTIR) used to detect different functional groups of metabolites. These techniques help to identify and quantify a variety of primary and secondary metabolites in the purified bioactive compounds (Pandey et al., 2016).

Different methods have been used for the extraction of bioactive compounds; these include microwave-assisted extraction (MAE) soxhlet extraction (SE) using different solvents as well as ultrasonic/ultrasound-assisted extraction (UAE). Other modern methods also in use include supercritical fluid extraction (SFE), solid-phase extraction (SPE), enzyme-assisted extraction, pressurized liquid extraction (PLE) or accelerated solvent extraction (ASE), and extraction assisted by a pulsed electric field. These recent methods are efficient in the removal of flavonoids from plant products. Current reviews have discussed in detail, the use of these modern methods for the extraction of essential bioactives from plants (Selvamuthukumaran and Shi, 2017; Chaves et al., 2020).

The MAE is one of the modern extraction methods that has received much patronage due to several merits such as a reduction in utilization of solvents, enhanced yield recovery, better selectivity, reproducibility, reduced operation time, and less sample manipulation (Alara and Abdurahman, 2019; Alara et al., 2019; Nour et al., 2021). MAE utilizes microwave energy for faster heating which results from a range of electromagnetic spectrums of light (300 MHz to 300 GHz) with short wavelengths usually between 1 cm^−1^ and 1 m^−1^. The combined effect of increased temperature within the extraction medium and the effect of microwave electromagnetic radiation on vibrations of both the extraction solvents and the analytes being extracted enhances the extraction yield (Ameer et al., 2017; Chaves et al., 2020). It is essential to note that the efficiency of the extraction process is dependent on several factors such as the temperature and particle size, solvent-solid ratio, as well as the nature of the solvent used for extraction.

MAE utilizes microwave energy for rapid heating which results from a range of electromagnetic spectrums of light (300 MHz to 300 GHz) with short wavelengths usually between 1 cm^−1^ and 1 m^−1^. The combined effect of increased temperature within the extraction medium and the effect of microwave electromagnetic radiation on vibrations of both the extraction solvents and the analytes being extracted enhances the extraction yield (Ameer et al., 2017; Chaves et al., 2020). It is noteworthy that the efficiency of the extraction process is dependent on several factors such as the temperature and particle size, solvent-solid ratio, as well as the nature of the solvent used for extraction. The extraction efficiency is dependent on factors such as the nature of the solvent, solvent–solid ratio, temperature, as well as particle size. To enhance the extraction process, optimization of modern extraction methods is required. Synergistic effects could be obtained if there is a combination of the above novel extraction methods. Tsamo et al. (2020) used an ultra-performance liquid chromatographic system with a PDA detector coupled to a mass spectrophotometer detector (UPLC-qTOF-MS) to identify phenolic compounds in the seeds of Kersting’s groundnut. A total of 57 potential compounds were identified, among which were flavonoids; catechin, gallocatechin, quercetin, rutin, naringin, kaempferol, 7-rutinoside, and eriodictyol 7-rutinoside.Gallocatechin is known to enhance lipid metabolism and aids the prevention of metabolic syndrome as has been reported in some other legume seeds such as pea and lentils (Mirali et al., 2017; Jha et al., 2019; Tsamo et al., 2020). **Table 6** shows different modern methods used in extraction of bioactive components of plants, including seeds.

**Table 6:** Bioactives from legumes and their extraction conditions.

Bioactive compound	Extraction method	Plant source	Extraction conditions	References
Phenolic phytochemicals	MAE	*Phaseolus vulgaris* (L.).	Effective extraction of polyphenols at a temperature of 150°C using 50% ethanol	(Sutivisedsak et al., 2010)
inositols and α-galactooligosaccharides	Optimized MAE	Mung bean (Vigna radiata)	0.5 g dry sample, 2 cycles of 3 min, 50°C, 10 mL 50:50 (ethanol: water, v: v), resulted in extraction of bioactive carbohydrates between 74.1 and 104.2 mg.g−1 dry sample	(Carrero-Carralero et al., 2018)
inositols,α-galactooligosaccharides (GOS)	MAS	Alfalfa (*Medicago sativa* L.) leaves and stems	Optimal extraction temperatures of 40°C (leaves), and 80° C (seeds) resulted in higher yields of inositols (2x) and α-GOS (7 x) with more Pinitol in leaves and stems (24.2–31.0 mg_g-^1^ and 15.5–22.5 mg_g-^1^, respectively) while seed extracts were rich in α-GOS, mainly in stachyose (48.8–84.7 mg_g1).	(Solarte et al., 2021)
Quercetin flavonoid	Sequential MAS	Red kidney bean	The extraction efficiency of quercetin was enhanced yielding 35.8 mg quercetin/g kidney bean	(Aghajanian et al., 2020)
Saponins	UAE	red lentils (*Lens* *culinaris*)	The ethanol extraction efficiency of total saponin content in red lentil seeds was increased (11 g 100 g^-1^).	(Del Hierro et al., 2018)

MAE, Microwave assisted extraction; UAE, Ultrasound-assisted extraction.

With the advancement in sequencing techniques and omics technologies such as genomics, proteomics, transcriptomics, metabolomics, and the genome editing tools such as the CRISPER-Cas9 or TALEN, underutilized legumes can be genetically improved for better utilization and acceptance as these legumes are currently faced with some production constraints such as high antinutritional factors in the seeds which reduce the bioavailability of minerals, prolonged cooking time due to hardness of the seed coat of some pulses, and photoperiod sensitivity which also affects tuberization in tuberous legumes. The utilization of plant-based functional foods can be enhanced by the use of innovative technologies for the extraction and microencapsulation of bioactive compounds using novel technologies in metabolomics (Nayak et al., 2021; Pattnaik et al., 2021). Metabolomic studies can be used to identify rich value-added compounds from different parts of underutilized legumes such as the seeds, leaves, stems, or tubers, listing the main bioactive metabolites identified and the factors affecting their production. Metabolomics finds its usefulness in the identification of metabolites after the bioactives have been extracted using one of the modern methods discussed above. It highlights the expressions and changes of metabolites, as well as their interactions and resulting phenotypic traits in plants subjected to harsh environmental conditions. Under such stress, plants must adapt their metabolomic pathways to maintain metabolic homeostasis, a process referred to as acclimation (Joshi et al., 2021; Makhumbila et al., 2022).

Chen et al. (2020) studied the metabolomic profile of common bean and identified major findings related to amino acids, flavonoids, isoflavonoids, purines, and proline metabolism. These pathways enhanced the plant’s potential for defense against pathogens like *Fusarium solani* (FS). The study combined RNA sequencing and metabolomics techniques to investigate changes in gene expression and metabolic processes in common bean infected with FS. The results showed that metabolic pathways were enriched, leading to an increase in metabolites involved in plant defense response. Infected common bean seedlings responded with modifications to their cell walls, the generation of reactive oxygen species, and a synergistic hormone-driven defense response. The study also found that infected plants induced energy metabolism, nitrogen mobilization, accumulation of sugars, and arginine and proline metabolism (Chen et al., 2020; Makhumbila et al., 2022). Reliable software tools such as GCMS, LC-MS, and NMR are required to analyze the vast amounts of data generated by metabolomic technologies. These tools should be capable of visualizing, detecting peaks, normalizing/transforming sample data, annotating, identifying, quantifying, and statistically analyzing targeted and untargeted metabolite variations using algorithms for univariate and multivariate analysis (Sun and Weckwerth, 2012; Junot et al., 2014; Makhumbila et al., 2022). There are now several metabolomic pathway databases available online that group metabolites with similar functions. These databases include the Kyoto Encyclopedia of Genes and Genomes (KEGG), Cytoscape, MapMan, and iPath, which are relevant to plants. Cytoscape is an open-source software platform used to visualize complex networks and integrate them with any type of attribute data. MapMan is a user-driven tool that displays large datasets onto diagrams of metabolic pathways while iPath is a relevant tool for plants (Fukushima and Kusano, 2013; Patel et al., 2021).

With the advent of Next-generation Sequencing (NGS), the cost of sequencing has plummeted, making it possible to sequence large and complex genomes in a shorter period (Hamilton and Robin Buell, 2012; Kumar et al., 2021). Several whole genome sequencing studies are underway for some underutilized crops, and some have been completed. Once these sequences are available, they can be applied for in-depth structural and functional genomic studies to characterize and annotate the genes. Furthermore, the availability of the whole genome sequence will accelerate the development of genetic linkage maps of genomic regions that control particular traits of the plant, as well as the accumulation of bioactive compounds. The coupling of sequencing technologies with bioinformatics and high-through put phenotyping techniques genomic studies and bioinformatics tools could facilitate the improvement of the genetic pathways for the production of bioactive compounds and identification of genes that regulate essential agronomic traits relevant to the quality of NULs (Mochida and Shinozaki, 2011; Steinwand and Ronald, 2020; Kumar et al., 2021). Utilization of various genomics approaches such as genome-wide association studies (GWAS); marker-assisted selection (MAS) and genomic selection (GS) have been used to identify useful markers linked to nutritional traits and bioactives in various crops. For instance, a study on 94 chickpea genotypes from a diverse population using GWAS resulted in the identification of eight single nucleotide polymorphisms (SNPs) associated with Fe and Zn content in chickpea seeds (Diapari et al., 2014), while two closely associated SNPs markers for Fe and Zn were identified by GWAS in lentils (Khazaei et al., 2017). Following the identification of SNPs linked to these trace elements, marker-assisted selection can be applied for the introgression of these traits into underutilized legumes through the process of biofortification. This is particularly relevant in the current global pandemic as Zn is known to be an immune booster. Shreds of evidence have shown that Zn deficiency increases the risk of infectious diseases, autoimmune disorders, and cancer (Roohani et al., 2013; Haase and Schomburg, 2019; Wessels and Rink, 2020; Wessels et al., 2020). Although most of the NULs especially the African yam bean are good sources of Zn and Fe, the levels of these elements could be enhanced through biofortification. Legumes with high Fe and Ze levels could be harnessed to boost the immunity of risk groups mainly the elderly and patients with inflammatory or autoimmune diseases. With the use of modern breeding methods, an international organization located in Malaysia, Crops for the Future is spearheading research on some underutilized species such as bambara groundnut and winged bean to enhance food and nutritional security.

Omic technologies should be employed to enhance the functional components of NULs towards ensuring food and nutritional security. The use of transcriptomic analysis for the identification of regulatory genes in biochemical pathways can assist researchers to gain significant insight into the functional mechanisms of plant’s biosynthetic pathways especially those involved in secondary metabolite synthesis. The transcriptomic analysis is usually carried out to study gene expression in plants. This is done using microarray technology or RNAseq analysis. Available transcriptome data of some model legumes could be applied to study NULs when the transcripts have been obtained as has been reported for *Medicago trunculata* using theCTDB (RNASeq) and MtGEA (Mocroarray) (Garg and Jain, 2013). Integrated use of omics technologies methods to enhance the nutrient potential of any crop, could influence nutritional security if applied in food processing and formulations (Tian et al., 2016; Nayak et al., 2021). If this is not done the rich bioactive compounds inherent in most of these indigenous legumes will remain unknown and untapped. Molecular studies should therefore be used for genetic dissection of antioxidant activities in NULs and nutrient-related traits. This has not received much research attention thus far. Apart from enhancing the nutritional contents of these legumes, omics technologies coupled with genome editing could aid the reduction of antinutrients like oxalate and phytic acid which affects the bioavailability of vital minerals, thereby enhancing human health.

TThe CRISPR/Cas 9 method of genetic manipulation in plants has gained a lot of attention and acceptance for crop improvement because it is straightforward, adaptable, and accurate (Bhowmik et al., 2021). It would be very useful for enhancing genetic improvement in NULs. This technology is constantly evolving and has a wide range of applications, such as producing knockouts, precise modifications, multiplex genome engineering, or controlling gene expression (Arora and Narula, 2017; Baloglu et al., 2022). CRISPR/Cas9 relies on two key components: a Cas9 endonuclease and a guide RNA (gRNA) consisting of two small RNA molecules: the CRISPR RNA (crRNA) which is a 20-nucleotide sequence that matches the target DNA, and the transactivating crRNA (tracrRNA), which serves as a binding scaffold (Bhowmik et al., 2021). The CRISPR/Cas9 technology is highly useful for improving genetic traits in plants. Though it has been utilized in gene editing of known legumes such as soybean, cowpea, and the model legume *Medicago trunculata* (Curtin et al., 2018; Al Amin et al., 2019; Bao et al., 2019; Ji et al., 2019; Bao et al., 2020; Juranic et al., 2020; Chen et al., 2020; Wolabu et al., 2020; Bhowmik et al., 2021), very few attempts have been made on genome editing in underutilized legumes. The bottleneck has been in getting the whole genome sequence of the underutilized legumes.

Meng et al. (2017) developed an efficient CRISPR/Cas9 system for inducing targeted mutations in the MtPDS gene in the model legume *M. truncatula*. Among the 309 T0 transgenic plants, 32 displayed the albino phenotype. To determine if the albino phenotype was due to the targeted mutation, 16 out of the 32 transgenic plants were randomly selected for sequence analysis. Results revealed that all the albino plants had mutations at the targeted site of the MtPDS gene. These findings were supported by Baloglu et al. (2022). Efforts are ongoing by the African Orphan Crops Consortium (AOCC) through a network of international to regional public-private partnerships and collaborators, to generate genomic sequences of some underutilized legumes (Faba bean, Mungbean, Bambara groundnut, Marama bean, Mungbean, and *Lablab purpureus*). The complete genome sequence of Mungbean published in 2014 permitted genomic research and molecular breeding of mung bean (Kang et al., 2014; Baloglu et al., 2022). Schafleitner et al. (2015) evaluated agronomic traits in 1481 Mungbean collections based on the availability of its whole genome sequence. This paves the way for genome editing to be used for the genetic improvement of this species to enhance its yield, nutritional content, and resistance to diseases. The lack of whole genome sequences in most of these beneficial underutilized legumes poses challenges to their improvement using CRISPER/Cas9. Despite the absence of a genome reference sequence for Faba bean, significant advancements have been made in genetic and genomic resources to aid molecular breeding. The robust synteny shared with the model legume *M. trunculata* allows for the use of omic technologies like transcriptomics and comparative genomics. These methods help identify single-nucleotide polymorphisms (SNPs), develop high-density consensus genetic maps, and predict the candidate genes responsible for various desirable traits. Bhowmik et al. (2021) discussed these approaches in their study. The genome editing technology no doubt has the potential to enhance crop improvement in various ways. However, some individuals are against its global acceptance pushing for regulations on its use. Nevertheless, genome editing technology differs from genetic engineering or modification which requires novel genes to be inserted into another organism of a different species. Across many countries and regions in the world, different regulatory approaches are being sought. In Africa, the National Biosafety Management Agency of Nigeria released the first gene editing guidelines which paves the way for its utilization in the improvement of economic crops in the country. Other African governments should take a cue from Nigeria and develop a regulatory framework for gene editing.

## 8 Conclusion

There is no doubt that underutilized legumes are rich sources of micronutrients and bioactive compounds with a great capacity to achieve zero hunger of the Sustainable Development Goals (SDG) by 2030. The rich bioactive compounds inherent in underutilized legumes which are yet to be tapped have great health benefits for man. Most phytochemicals in legumes are regarded as antinutritional components as they have no nutritional value. However, recent studies have shown that these non-nutrients such as tannins, glycosides, and saponins possess hypocholesterolemic and anticarcinogenic activity while flavonoids possess antioxidant activities which are essential for scavenging reactive oxygen species which cause oxidative stress in diseased conditions. If the abundant bioactive compounds in these underutilized legumes are identified and employed as therapeutics or used in the development of functional food products, it will greatly enhance human health, reduce the over utilization of the common legumes as well as help to increase food, protein, and nutrition security in Africa. The renewed efforts in this direction will be to evolve strong research and development between industries (pharma and foods) and the academia/research for appropriate food-based or pharmaceutical product developments. Thus, incorporating NULs rich in bioactive compounds into the diet of man will boost achieving the Sustainable Development Goal 3 of the United Nations on good health and well-being.

## Author contributions

JP and OO – conceived the idea, wrote the first draft, searched the literature, and reviewed the manuscript. OA, LO, TA, MA, AO, SD, and AA – contributed to the writing and review of the manuscript. PA, SO, MA, and CO – contributed to the writing. JP – edited, fine-tuned, and approved the final draft. All authors contributed to the article and approved the submitted version.
